# Tinnitus in Normal-Hearing Individuals: Is Outer Hair Cell Dysfunction the Mechanism?

**DOI:** 10.3390/jcm14155232

**Published:** 2025-07-24

**Authors:** Theognosia Chimona, Maria Vrentzou, Emmanouel Erotokritakis, Eleni Tsakiraki, Panagiota Asimakopoulou, Chariton Papadakis

**Affiliations:** 1 ENT Department of Chania General Hospital, 73133 Chania, Greece; vrentzou.m@gmail.com (M.V.); elen.tsak30@gmail.com (E.T.); giwta_asima@hotmail.com (P.A.); chpap@hotmail.com (C.P.); 2 Independent Researcher, 19400 Koropi Attiki, Greece; erotokritakis@hotmail.com

**Keywords:** tinnitus, normal hearing threshold, tinnitus analysis, otoacoustic emissions, distortion product otoacoustic emissions

## Abstract

**Background/Objectives:** Cochlear “injury” is thought to be a significant cause of tinnitus in patients with hearing loss. Interestingly, individuals with normal hearing may also experience tinnitus. This study evaluates otoacoustic distortion product emissions (DPOAEs) in individuals with normal hearing who experience tinnitus perception. **Methods:** In this prospective study, the tinnitus group (TG) consisted of 34 subjects with tinnitus (four unilaterally) and normal hearing (threshold ≤ 25 dBHL at 0.25–8 kHz). The control group (CG) comprised 10 healthy volunteers (20 ears) without tinnitus and normal hearing. Medical history was recorded, and all participants underwent a complete otolaryngological examination, pure tone audiometry, and DPOAE recording (DP-gram, L1 = 55 dB, L2 = 65 dB, for F2: 619–10,000 Hz). Moreover, participants in the TG completed a detailed tinnitus history (with self-rated loudness scoring) and the Tinnitus Handicap Inventory (Greek-version THI-G) and underwent tinnitus analysis. **Results:** The recorded mean DPOAE values during the DP-gram of the CG were significantly larger in amplitude at low (*t*-test, Bonferroni-corrected *p* < 0.09) and high frequencies (*t*-test, Bonferroni-corrected *p* < 0.02) compared with the TG. Tinnitus assessment showed tinnitus pitch matching at the frequency area in the DP-gram, where the acceptance recording criteria were not met. There were no statistically significant differences in tinnitus onset, self-rated loudness scores of >70, and severe disability (THI-G > 58) for TG subjects in whom DPOAEs were not recorded at frequencies of ≤1000 Hz. Participants with abnormal DPOAEs at around 4000 Hz had tinnitus of sudden onset and severe disability (THI-G > 58). Finally, those with pathological recordings of DPOAEs at ≥6000 Hz had gradual onset tinnitus (Pearson Chi-square test, *p* < 0.05). **Conclusions:** DPOAEs in normal hearing individuals with tinnitus show lower amplitudes in low and high frequencies compared with normal hearing individuals without tinnitus. The tinnitus matched-frequency coincided with the frequency area where DPOAEs were abnormal.

## 1. Introduction

Tinnitus, the involuntary, subjective perception of noises or tones without the presence of external or internal auditory stimuli, is a prevalent and impactful condition. In most cases, it starts from acquired hearing loss and persists when this loss is combined with distinct neural changes in the auditory and other brain neural networks [1]. The prevalence of tinnitus is reported to range from 10% to 20% of adults, with higher incidence in males and older individuals [2,3]. Tinnitus represents one of the most common and challenging otological problems in everyday clinical practice. It may manifest with accompanying symptoms that further impair the patient’s quality of life (QoL), such as hearing loss, depression, anxiety, insomnia, and hyperacusis [4]. The impact of tinnitus on QoL does not necessarily relate to its intensity or psychoacoustic characteristics. For some, tinnitus is not considered a significant problem in their routine, while for others, it is a devastating symptom [5].

Studies have demonstrated a strong link between hearing loss and tinnitus, showing significant correlations between the frequency and intensity of tinnitus and hearing thresholds [6]. Tinnitus can have various causes, including temporary conditions like inflammation of the middle and outer ear or, more frequently, permanent issues such as presbycusis, chronic noise exposure, ototoxic medications, head trauma, otosclerosis, Ménière’s disease, otologic surgeries, meningitis, cerebellopontine angle tumors, and central nervous system (CNS) disorders [7]. Additionally, tinnitus has been attributed to systemic disorders and conditions such as autoimmune diseases, depression, sleep deprivation, sleep apnea, dyslipidemia, hypertension, hyperglycemia, and smoking [3,8,9,10]. Interestingly, even individuals with normal hearing may experience tinnitus.

The most common causes of tinnitus are noise-induced hearing loss and presbycusis [5]. Both conditions present with hearing loss at higher frequencies. They lead to the destruction or dysfunction of cochlear sensory receptors, specifically inner and outer hair cells (IHCs and OHCs). The primary function of OHCs is to mechanically amplify sound vibrations on the cochlear membrane, acting as a “cochlear amplifier” and being responsible for precise frequency discrimination. With the stimulation of cochlear hair cells, mechanosensitive ion channels open, depolarizing the hair cells and causing them to release neurotransmitters onto the dendrites of spiral ganglion neurons. These neurons’ axons then transmit auditory information to the brain. Exposure to loud sounds can damage cochlear hair cells, resulting in permanent hearing threshold elevations. Even moderate sound intensities can cause a “temporary” shift in hearing threshold that eventually returns to pre-exposure levels. Although hearing thresholds may only be temporarily affected, this damage can lead to permanent functional hearing deficits [1]. These peripheral changes can trigger plasticity in central auditory and non-auditory neural networks, resulting in the perception of tinnitus [1]. Although peripheral dysfunction and hearing loss are significant causes for tinnitus perception, some individuals suffering from tinnitus do not have measurable hearing deficits in standard audiological tests [11,12]. It is reported that among all tinnitus patients, 7–10% have normal pure tone audiograms for the frequencies of 125–8000 Hz, although high-frequency hearing loss may occur at frequencies above 8000 Hz [13].

A detailed history and clinical examination are crucial in distinguishing between benign and dangerous tinnitus causes and identifying potential treatment options. The initial assessment should include identifying tinnitus characteristics that justify urgent management, i.e., a pulsatile nature, tinnitus accompanied by neurological abnormalities, unilateral location, and asymmetric hearing loss [4]. An important step in recording patients’ histories is their medications, as a long list of pharmaceutical substances has been implicated in the onset of tinnitus [7]. Questionnaires that highlight the degree of disability and the impact of tinnitus on quality of life (QoL) can also be valuable tools, as they may serve as measures for evaluating therapeutic interventions [14,15,16].

Research on tinnitus patients with normal hearing has been conducted to explore clinical characteristics and results from other audiological and electrophysiological tests [11,12,13,17]. Otoacoustic emission (OAE) recording, discovered by David Kemp in the 1990s, allows the evaluation of OHCs’ function [18,19]. OAEs are sound signals recorded in the outer ear canal when the tympanic membrane receives vibrations transmitted back from the cochlea through the middle ear. These vibrations are produced as by-products from OHCs, the “cochlear amplifier” [19,20].

OAEs are present when Corti’s organ is in near-normal condition and can only be recorded when the middle ear functions normally [20]. Transient-evoked OAEs (TEOAEs) and distortion-product OAEs (DPOAEs) are used in clinical practice. A significant advantage of TEOAEs and DPOAEs is that they are quick, objective methods requiring no active participation from the examinee. Thus, they have both been implicated in newborn hearing screening protocols. Furthermore, they are important tools for the diagnosis of auditory neuropathy spectrum disorder, where they are normally recorded, but the Auditory Brainstem Response is abnormal, and individuals present with various patterns of audiometric configuration. DPOAEs are particularly valuable, as they are frequency-specific [17,18]. DPOAEs arise when two pure tones of different frequencies, F1 and F2 (F2 > F1, with a ratio of F2/F1 = 1.2), are applied to the outer ear canal with intensities L1 and L2, respectively. They are produced and recorded in almost all normal ears and have low intensity, from –20 dBSPL to 15 dBSPL. Many by-products are produced, but 2F1-F2 has the highest amplitude and is the one that is routinely recorded in practice [20,21].

The research on recording evoked OAEs, particularly DPOAEs, in tinnitus patients has revealed varying results. Shiomi et al. demonstrated reduced DPOAE amplitude in nine tinnitus patients, especially at frequencies between 4000 Hz and 7000 Hz [22]. Other researchers have shown that DPOAE recordings were abnormal in 13 tinnitus patients with normal hearing and that there was a non-linear relationship between hearing threshold and DPOAE amplitude [18]. Conversely, Sztuka et al. demonstrated significantly higher DPOAE amplitude in tinnitus patients without hearing loss compared with non-tinnitus individuals, concluding that tinnitus may be caused by increased OHC motility due to reduced efferent fiber activity rather than OHC deficiency [23]. Moreover, in a recent study, the authors thereof report that OHC dysfunction is not necessary for tinnitus perception; thus, they conclude that other changes in the auditory pathway may be responsible for tinnitus occurrence [12]. The inconsistency in findings may be attributed to heterogeneity of tinnitus etiologies, study population differences, variability in hearing thresholds, hidden hearing loss, interindividual variability in DPOAE amplitudes, and different methodologies [24,25]. Additionally, the averaging of DPOAE results across patients may hide meaningful subgroup differences, such as frequency-specific dysfunction or side-specific tinnitus [26].

Although the role of DPOAEs in individuals with normal hearing and tinnitus is not fully clear, some researchers consider DPOAEs a useful tool for assessing cochlear pathology, particularly at an early stage of OHC dysfunction, before hearing-threshold shifts become clinically apparent. This study aims to evaluate the amplitudes of DPOAEs in individuals with normal hearing and tinnitus and to investigate the correspondence between pathological DPOAE frequencies and their tinnitus frequency. Secondary questions of this study were (a) whether tinnitus quality corresponded to abnormal DPOAE recordings at specific frequencies and (b) whether there were tinnitus-related characteristics from the patient’s history that corresponded to abnormal DPOAE recordings at specific frequencies.

## 2. Materials and Methods

### 2.1. Participants

This study was designed as a prospective comparative analysis to evaluate DPOAE recordings in two groups: individuals with normal hearing and tinnitus (the tinnitus group, TG) and a control group of normal hearers without tinnitus (CG). The research was conducted in the audiology lab of the ENT department at Chania General Hospital, spanning from June 2023 to January 2025. The TG consisted of 34 individuals who had primary subjective tinnitus but normal pure-tone thresholds, while the CG included 10 healthy volunteers with no history of any otological disorder. The TG participants were recruited from the outpatient clinic of the ENT department of Chania General Hospital, and the CG consisted of volunteer staff (doctors and nurses) from the same institution. Upon inclusion in this study, all participants gave written informed consent for all procedures. The inclusion criteria for the TG were (a) a hearing air conduction threshold on the pure-tone audiogram for frequencies of 250–8000 Hz ≥ 25 dBHL, air-bone gap ≤ 5 dB; (b) unilateral or bilateral tinnitus lasting for more than 3 months; (c) age of >18 years; and (d) normal findings on magnetic resonance imaging of the temporal bones. For the CG, the inclusion criteria were (a) a hearing threshold on the pure-tone audiogram for frequencies of 125–8000 Hz ≥ 25 dBHL and (b) age of >18 years. The exclusion criteria were (a) pulsatile tinnitus; (b) disorders with fluctuating hearing loss and tinnitus (e.g., autoimmune diseases, Ménière’s disease); (c) active ear infection; (d) abnormal tympanograms; (e) neurodegenerative diseases; (f) psychiatric disorders or use of antipsychotic medication; (g) ototoxic medication, i.e., chemotherapy or regular use of aspirin or furosemide; (h) individuals who did not speak/read the Greek language; and (i) refusal to give written consent.

### 2.2. Clinical Examination and Questionnaires

This study was conducted in a separate scheduled appointment for each participant. All participants underwent complete otorhinolaryngological examination. Additionally, in the TG, a tinnitus history was recorded [type of onset, duration, self-assessment of annoyance (score 1–100), self-assessment of intensity (score 1–100), situations that worsen or improve tinnitus, etc.]. Finally, the TG participants completed the Greek validated version of the Tinnitus Handicap Inventory (THI-G) questionnaire [16]. The THI is a 25-item self-assessment questionnaire designed to determine the perception of disability severity due to tinnitus. Each answer is scored separately as follows: yes = 4, sometimes = 2, no = 0. A total score below 16 indicates “slight disability,” a score of 18–36 indicates “mild disability,” 38–56 corresponds to “moderate disability,” a score of 58–76 indicates “severe disability,” and a score of 78–100 is classified as “catastrophic disability” [14,16]. The THI is divided into three subscales. The functional subscale, which consists of 11 items, assesses the impact of tinnitus on mental, social, occupational, and physical functioning. The emotional subscale contains 9 items and reflects an individual’s emotional responses to tinnitus, such as anger, frustration, irritability, and depression. The catastrophic subscale includes 5 items and is designed to reveal feelings of desperation, lack of control, and an inability to cope with the condition [16].

### 2.3. Audiometric Examinations

Participants in both groups underwent tympanometry, pure-tone audiometry, and DPOAE recording. Each ear was evaluated separately. Pure-tone audiometry was performed in a soundproof booth with the Clinical Audiometer AC-40 by Interacoustics, Middelfart, Denmark. Air conduction thresholds were evaluated by using TDH-49 headphones, Middelfart, Denmark, at the frequencies of 250–500–750–1000–2000–3000–4000–6000–8000 Hz. Bone conduction thresholds were evaluated by using a B71 bone conductor at the frequencies of 250–500–1000–2000–4000 Hz. DPOAE recording took place in a quiet room using the EclipseDPOAE20 platform, version EP25 by Interacoustics, Middelfart, Denmark. The software graphically presented the recording as a DP-gram, i.e., a graph of the amplitude of the DPOAEs as a function of frequency F2. Stimulus parameters were tones’ intensity, L1 = 65 dBSPL and L2 = 55 dBSPL, respectively, and the frequency ratio between F1 and F2 was set at F2/F1 = 1.22 (Table 1). The distortion product frequency measured was 2F1-F2. The frequencies, F1 and F2, tested in the DP-gram for each participant are shown in Table 1.

The criteria for acceptable DPOAE recordings included a sound-to-noise ratio (SNR) of > 7 dB, DPOAE intensity of > −10 dB SPL, ambient noise intensity of < −10 dB SPL, and recording amplitude within the normal range for the participant’s sex. Each recording was performed twice, and the best one was selected, i.e., the recording where the acceptance criteria for normal DPOAEs were met in most of the examined frequencies.

In all participants in the TG, tinnitus analysis was conducted using the Tinnometer by MedRx, byMedRx, Inc., Largo, FL, USA. which recorded tinnitus frequency (pitch matching) and tinnitus intensity (loudness matching). The determination of tinnitus frequency was made with an accuracy of 1 Hz after selecting the frequency range that corresponded to the description of the participant’s tinnitus (i.e., “ringing,” “whistling,” “hissing,” “humming,” “cicadas”). At the end of the evaluation, tinnitus sound therapy was recommended to most of the TG participants via a Quick Response Code (QR code) generated by the Tinnometer, which was linked to an audio file.

### 2.4. Evaluated Parameters and Statistical Analysis

The evaluated parameters included (1) the intensity of recorded DPOAEs and the comparison of their mean values between the two study groups (TG and CG); (2) the qualitative analysis of tinnitus in relation to DPOAE recordings; and (3) the matching of specific frequencies of non-recorded DPOAEs to specific characteristics from the patient’s history, specifically: (a) the type of tinnitus onset (gradual or sudden), (b) self-assessed tinnitus intensity of >70 (on a scale from 0 to 100), and (c) severe disability according to THI-G results. Descriptive statistics were used for continuous variables, and a *t*-test (Bonferroni-corrected) was conducted to compare the mean values of DPOAE intensities between the two study groups (TG and CG). Normality of continuous variables was assessed using the Shapiro–Wilk test. The evaluation of tinnitus-specific characteristics related to DPOAE recordings was performed by using the Pearson Chi-square test. The statistical significance level was set at *p* < 0.05. Statistical analysis was conducted using the IBM SPSS Statistics for Windows, Version 26.0 (IBM Corp., Armonk, NY, USA).

## 3. Results

### 3.1. Primary Outcomes

**Figure 1:**
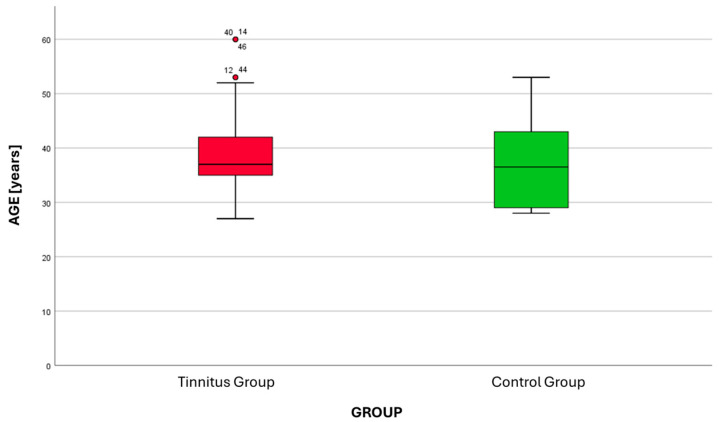
Box plot graph of age distribution in the tinnitus group (TG) and the control group (CG). Outliers for TG: 12, 44 (53 years), 14, 40, 46 (60 years); outliers for CG: none.

There was no statistically significant difference in the mean age of the participants between the two groups (*t*-test, *p* = 0.35). The TG had an average age of 37.8 years (SD ± 7.8), while the CG had an average age of 36.9 years (SD ± 8.3) (Figure 1).

In the TG, 30 participants reported experiencing bilateral tinnitus or tinnitus perceived at the center of the head, while 4 participants experienced unilateral tinnitus (three on the left and one on the right side). Additionally, two patients reported hyperacusis. Each ear was studied separately, resulting in a total of 64 ears in the TG and 20 ears in the CG. The TG participants’ responses on the THI-G yielded an average score of 42 (SD ± 21.9), with a minimum score of 4 and a maximum score of 94.

**Figure 2:**
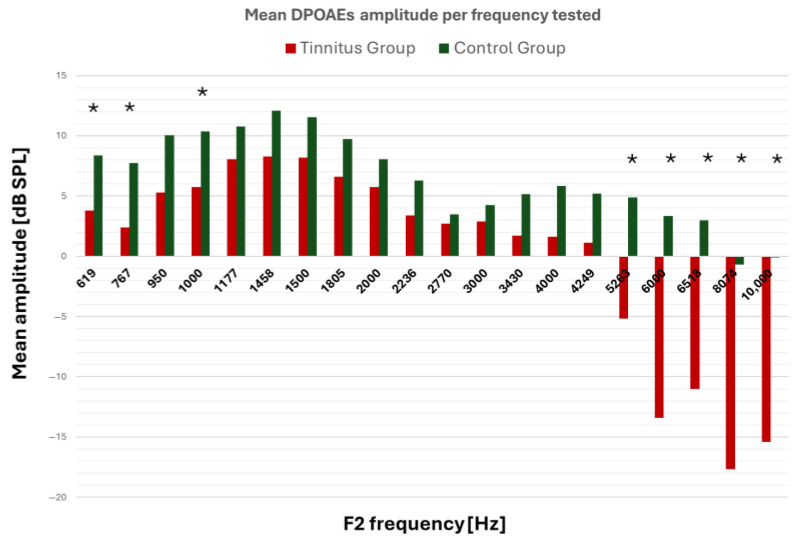
Graph of the recorded DPOAEs’ mean values for the tinnitus group (red bars) and the control group (green bars). The symbol * indicates values with significant statistical differences (*t*-test, Bonferroni-corrected, *p* < 0.05). Abbreviations: DPOAEs = distortion product otoacoustic emissions; F2 = the frequency in Hz at which the DPOAEs were measured; dB SPL = decibel sound pressure level.

Table 2 and Figure 2 present the recorded mean values of the DPOAEs for both study groups during the DP-gram procedure. The comparison of DPOAE amplitudes between the TG and the CG revealed significantly reduced responses in the tinnitus group at several key frequencies, even after Bonferroni correction for multiple comparisons.

Specifically, statistically significant differences were observed at F2 frequencies of 619 Hz, 767 Hz, 1000 Hz, 5263 Hz, 6000 Hz, 6518 Hz, 8074 Hz, and 10,000 Hz (*p* < 0.05, Bonferroni-corrected), with consistently lower DPOAE amplitudes in the tinnitus group. These results suggest frequency-specific outer hair cell dysfunction in individuals with tinnitus, affecting both low- and high-frequency cochlear regions. Importantly, this dysfunction was present despite normal audiometric thresholds, highlighting the value of DPOAEs in detecting subclinical cochlear alterations that may underlie tinnitus perception. The largest differences were noted in the high-frequency range (6000–10,000 Hz), which is often associated with “hidden” cochlear damage.

A post hoc analysis using the G*power platform, version 3.1.9.7, revealed a statistical power of 82% for the study sample, with a significance level of α = 0.05 and an effect size of d = 0.8.

**Figure 3:**
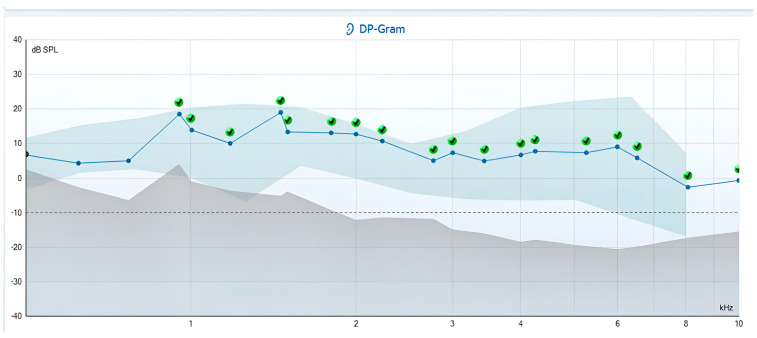
DP-gram of the left ear in a 45-year-old woman. She had been experiencing bilateral tinnitus for the past 12 years, described as a “buzzing” sound perceptible 80% of the day time. Her THI-G score was 38; she self-rated the intensity of her tinnitus at 30 (on a scale of 1–100). Tinnitus analysis revealed a left-ear tinnitus pitch match at 790 Hz, with an intensity of 49 dBSPL and a minimum masking level (MML) of 68 dBSPL. On the DP-gram, no DPOAEs were recorded at frequencies of F2 619 Hz, 767 Hz, and 950 Hz.

**Figure 4:**
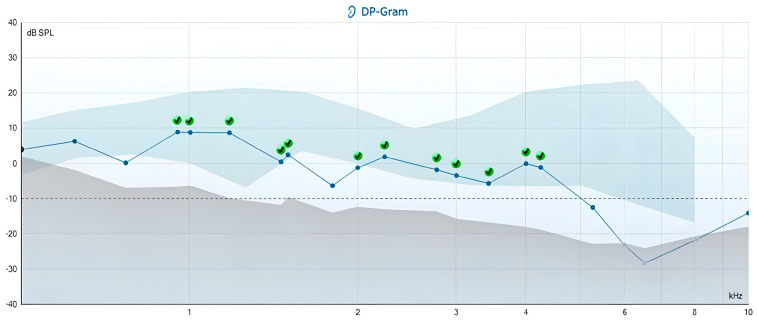
DP-gram of a 53-year-old woman with a sudden onset of tinnitus in the left ear, which she described as a “blowing” sound. Her THI-G score was 32; she self-rated the intensity of her tinnitus at 60 (on a scale of 1–100) and reported that it was perceptible 40% of the time. Tinnitus analysis revealed a tinnitus pitch match at 7142 Hz, with an intensity of 45 dBSPL and a minimum masking level (MML) of 53 dBSPL. DPOAEs did not meet the acceptance criteria at frequencies of F2 619 Hz, 767 Hz, and 1805 Hz, as well as frequencies greater than 5263 Hz.

Tinnitus analysis in the TG indicated that the frequency of tinnitus aligned with the frequency range on the DP-gram, where the recording criteria were not met in 29 participants, accounting for 85.3% of the group (see examples in Figure 3 and Figure 4). For the remaining participants, a tinnitus analysis could not be conducted because their tinnitus was not perceptible during the evaluation.

### 3.2. Secondary Outcomes

In participants with tinnitus described as “whistling,” the DPOAEs did not meet the recording criteria for frequencies of 6 kHz and above (26 participants, 49 ears). Among those who characterized their tinnitus as “cicadas” (5 participants, 9 ears), the DPOAEs did not meet the recording criteria for frequencies around 4 kHz. Additionally, 3 participants (6 ears) who did not have DPOAEs recorded at lower frequencies experienced tinnitus as a “humming” sound, which was more intense and of longer duration, even in noisy environments.

Furthermore, as shown in Table 3, there was no statistically significant difference in the mode of tinnitus onset among the TG subjects who had abnormal DPOAEs at frequencies ≤ 1 KHz (as determined by Fisher’s exact and Chi-square tests, *p* > 0.05). Participants who exhibited pathological DPOAE recordings at around 4 KHz were found to have sudden tinnitus onset (tinnitus perceived for the first time abruptly, within a 24 h period) and reported a high degree of disability with a THI-G score of over 58 (Fisher’s exact Chi-square test, *p* < 0.05). Conversely, those with pathological DPOAE recordings at frequencies of ≥6 KHz experienced a gradual onset of tinnitus (tinnitus with progressive development over a period of longer than 24 h, presenting a slowly increasing awareness or intensity over days, weeks, or months) (Fisher’s exact Chi-square test, *p* < 0.05).

## 4. Discussion

The results of this study showed pathological DPOAE recordings in participants in the TG with tinnitus and normal hearing at both low and high frequencies compared with the CG. Furthermore, in most TG participants, the tinnitus frequency identified through pitch matching matched the abnormal DPOAE frequencies on the DP-gram.

Tinnitus has been extensively studied to identify laboratory markers that correlate it with specific pathologies of the auditory system. Since the pathophysiology of “primary or idiopathic” tinnitus (i.e., those associated with or without sensorineural hearing loss, as opposed to “secondary” tinnitus caused by other pathological conditions independent of hearing loss) is not yet fully understood, various laboratory testing protocols have been proposed. Given that cochlear mechanisms may play a significant role in the generation and perception of tinnitus, evaluation of inner ear function is fundamental [7]. Otoacoustic emissions offer a non-invasive, objective method for assessing cochlear OHC function. Since their initial characterization by Kemp as “cochlear echoes,” otoacoustic emissions have proven valuable across a range of audiological applications, including neonatal hearing screening, diagnosis of auditory neuropathy, and differentiation of cochlear versus retrocochlear dysfunction [20].

The objective of this study was to investigate the characteristics of DPOAE recordings in tinnitus sufferers with normal hearing (TG) in comparison with DPOAE recordings from individuals with normal hearing without tinnitus (CG). The average age between the groups did not differ significantly, reducing confounding effects from age-related auditory degeneration. The majority of TG participants reported experiencing tinnitus in both ears or at the center of their heads, with only four participants reporting unilateral tinnitus. No participant presented with middle or outer ear pathology or a history of ototoxic drug exposure.

DPOAE amplitude values in the TG were significantly lower at both low and high F2 frequencies compared with those of the CG (Table 2), aligning with prior studies. Shiomi Y. et al. reported that the mean DP-gram of a group of nine individuals with tinnitus and normal hearing was significantly lower than that of normal individuals (*p* < 0.01) [22]. Later, in a study of 20 men with tinnitus and normal hearing thresholds aged 20–45 years, it was found that the mean total amplitude of DPOAEs in tinnitus patients was significantly lower than in individuals without tinnitus (*p* < 0.05) [27]. In this study, no correlation was found between the intensity of tinnitus and the amplitude of DPOAEs [27]. A 2008 study found reduced mean DPOAE amplitudes in tinnitus patients with normal hearing compared with non-tinnitus subjects (right ear: 3.63 vs. 1.95 dB SPL; left ear: 3.72 vs. 11.45 dB SPL) [17]. Karimiani et al. highlighted that DPOAE recordings at 10 kHz help determine differences in cochlear function between patients with and without tinnitus [25]. However, the reduced DPOAE levels at 2500, 5000, and 6298 Hz observed in patients with tinnitus and normal hearing suggest some damage to the OHCs that was not detectable with conventional pure tone audiometry [25]. Alshabory et al., in a larger cohort with normal pure tone thresholds (n = 50), found that 56% experienced bilateral tinnitus [28]. The DP-gram showed a greater mean amplitude in the control group compared with patients with tinnitus and normal hearing. The input/output study of DPOAEs at different frequencies (1, 2, 4, and 6 kHz) also showed higher amplitudes at all frequencies and various input intensity levels [28].

Nonetheless, findings in the literature are not consistent. Gouveris C. et al. clinically studied patients with acute onset of tonal tinnitus and found that an altered functional state of the OHCs appears in a selected high-frequency region of the cochlea in ears with acute tonal tinnitus and normal or nearly normal hearing thresholds [29]. Specifically, they found that ears with tinnitus showed relatively increased DPOAE amplitudes at high frequencies (4–6.3 kHz) compared with the group of healthy ears and relatively decreased DPOAE amplitudes at midfrequencies (1650–2400 Hz). Statistically significant (*p* < 0.01) increased mean DPOAE amplitude values were observed only at an F2 frequency of 4.9 kHz [29]. Sztuka et al., using a different protocol (e.g., L1 = L2 = 70 dB SPL, more recording points per octave), revealed significantly higher DPOAE amplitudes in the group of tinnitus patients without hearing loss and attributed these to increased OHC motility, potentially due to diminished efferent inhibition [23]. In their study, hyperacusis—present in 63% of participants—was linked to higher DPOAE amplitudes [23]. Acle-Cervera et al. reported lower mean signal-to-noise (S/N) ratios in tinnitus patients compared with controls, though these differences were not statistically significant (*p* > 0.05), leading to the conclusion that OHC dysfunction may not be necessary for tinnitus onset [12]. A recent study compared the mean amplitudes of DPOAEs in four groups of individuals with normal hearing: (a) a control group (n = 19), (b) tinnitus group (N = 15), (c) hyperacusis group (N = 10), and (d) tinnitus with hyperacusis group (N = 12). The results did not reveal significant differences in the mean amplitude of DPOAEs in individuals with tinnitus and/or hyperacusis and therefore do not support a peripheral mechanism or an interaction between peripheral and central mechanisms causing tinnitus or hyperacusis [30]. The authors acknowledged a limitation of their study: the small number of participants may have resulted in a non-significant difference in the amplitude of recorded DPOAEs between the groups [30].

In the early years of tinnitus research, the focus was primarily on changes in the peripheral auditory system, such as OHC loss, synaptopathy, and degeneration of type I spiral ganglion neurons [1]. Type I neurons are essential for auditory signal transmission, while type II neurons are primarily responsive to high-intensity stimuli and may contribute to tinnitus development [1]. However, as studies progressed, the theories began to shift toward recognizing the involvement of the central auditory system and the auditory cortex in tinnitus development [31]. Some researchers propose that tinnitus is the result of a mismatch between the expected “presentation” of sounds in the brain and the actual sensory input from a damaged cochlea [32].

We conclude that studying tinnitus through the function of outer hair cells (OHCs) using DPOAE recordings yields conflicting results. This is primarily due to the heterogeneity and diversity of tinnitus pathophysiology, the small sample sizes typically seen in related studies, variability in hearing thresholds, interindividual variability in DPOAE amplitudes, and different methodologies [24,25]. In some patients with tinnitus who have normal hearing, OHC damage can be detected through abnormal DPOAE recordings even before any shift in hearing threshold is observed in the tonal audiogram. However, for those experiencing synaptopathy, degeneration of type I spiral ganglion neurons, neuronal dysfunction, and alterations in neural synchrony and tonotopic organization, DPOAE recordings are not the preferred assessment method.

A noteworthy finding was that in 29 TG participants (85.3%; 55 ears), the tinnitus frequency coincided with DP-gram regions lacking valid DPOAE recordings. Two patients (four ears) were unevaluable due to imperceptible tinnitus during testing. This finding is supported by other studies [6,25]. In contrast, Shiomi et al. found mismatched regions in 73.3% of cases [22]. These results suggest that damage to OHCs may demonstrate tonotopicity in the emergence of tinnitus; nevertheless, this cannot imply a causal relationship, for, as discussed earlier, central neural processes also play a significant role [31,32]. Traditionally, tinnitus analysis—which involves evaluating its frequency and intensity (pitch and loudness matching)—is performed using a clinical audiometer in a soundproof booth or a quiet room. The process of measuring tinnitus frequency becomes simpler and achieves an accuracy of 1 Hz with the Tinnometer MedRx tinnitus meter, along with its corresponding software. This software offers additional options for adjusting rates and filters to ensure the best possible match. Patients receive a printed result that includes all evaluation details for each ear, such as frequency, intensity, MML (minimum masking level), and a QR code that links to an audio file for sound therapy.

In our study, the TG participants had an average total score of 42 on the THI-G questionnaire, which indicates “moderate disability.” Notably, one patient scored 94 and also experienced hyperacusis. This finding aligns with other studies that show a small percentage of tinnitus sufferers with normal hearing reporting severe disability based on their THI scores [28]. The THI questionnaire has been found to have high internal consistency, high convergent validity, good sensitivity, and the ability to distinguish between patients with different severities of tinnitus [14]. For these reasons, it is considered a useful tool in assessing therapeutic interventions in tinnitus patients.

Medical history analysis revealed distinct onset profiles. Participants with 4000 Hz tinnitus typically reported a sudden onset and high intensity, suggestive of acoustic trauma, even with no confirmed exposure. In contrast, tinnitus at ≥6000 Hz tended to develop gradually and intensify over time, resembling presbycusis without age-appropriate justification. This pattern may reflect subclinical cochlear insults or early degeneration preceding audiometric threshold shifts. Low-frequency tinnitus (≤1000 Hz) was generally reported as intense, audible in noisy environments, and requiring high masking intensity. The above characteristics were also statistically confirmed, as it was found that participants with pathological DPOAE recordings at around 4000 Hz had tinnitus of sudden onset, while those with pathological DPOAE recordings at 6000 Hz had tinnitus of gradual onset. There was no statistically significant difference between the severe and non-severe disability levels for those suffering from tinnitus with a pitch at ~4000 Hz or ≥6000 Hz (Table 3).

The advantages of the present study are (a) the performance of DPOAEs and the analysis of tinnitus by the same examiner each time and (b) the analysis of tinnitus with the help of the Tinnometer MedRx, which provided frequency detection accuracy of 1 Hz up to the value of 16,000 Hz, in contrast to the clinical audiometer, where the frequency-check “step” is of the order of an octave or a half-octave. Additionally, the software provided more sounds for pitch matching that more easily resembled tinnitus than the simple “tone” and “noise” traditionally used with the clinical audiometer.

The limitations of this study are (a) the small sample size of the TG—although many similar studies also have small numbers of participants, increasing the sample size could have enhanced the statistical power of this study; (b) the small size of the CG selected by healthy co-workers; (c) that the loudness of tinnitus was not studied in relation to DPOAEs, although in a study by Mokrian H. et al., no relationship was found between the loudness of tinnitus and the extent of OHC damage [27]—other factors than cochlear dysfunction may influence tinnitus loudness, such as dissociation between loudness perception and peripheral function due to central auditory gain, or psychological modulation of loudness; (d) no previous baseline DPOAE recordings were available for the TG, which should have helped with interindividual variability in DPOAE amplitude; (e) there is no possibility of long-term follow-up of the participants, as it has been shown that over the years, individuals with tinnitus and normal hearing develop more severe hearing loss, especially at high frequencies, compared with individuals without tinnitus [33]; (f) lack of objective electrophysiological markers [e.g., Auditory Brainstem Response (ABR) or Electrocochleography (ECoG)]; and (g) finally, a potential limitation in the inclusion criterion of a hearing threshold of ≤25 dB HL for frequencies of 250–8000 Hz. The threshold criterion could be set at ≤20 dB HL or 15 dB HL; however, finding a suitable sample could have posed a challenge.

Despite these limitations, this study highlights the potential of DPOAEs in detecting subtle cochlear dysfunction in tinnitus patients with normal hearing. Such micro-damages may lead to perceptual gaps that the central nervous system attempts to compensate for via neuroplastic mechanisms—potentially contributing to the tinnitus percept.

Suggestions for future research could include (a) studying a larger sample population to improve statistical power and draw reliable conclusions; (b) studying a separate group of individuals with tinnitus and hyperacusis; and (c) studying individuals with normal hearing and tinnitus in combination with DPOAEs (correlating the intensity of tinnitus with the recording of otoacoustic emissions) with electrophysiological tests (e.g., ABR, ECoG) to assess peripheral synaptopathy and central processing deficits, aiming to localize the origin of tinnitus more precisely across the auditory pathway.

## 5. Conclusions

Normal-hearing individuals with tinnitus show significantly lower mean DPOAE amplitudes in DP-gram recordings at some of the tested F2 frequencies compared with individuals with normal audiogram thresholds who do not complain of tinnitus. This suggests that individuals with normal hearing and tinnitus are likely experiencing dysfunction in the OHC compared with those in the control group. Analysis of tinnitus (via pitch matching) indicated that the tinnitus frequency fell within the F2 region, where DPOAEs did not meet the recording criteria.

A notable advantage in analyzing tinnitus among the TG participants was the use of specialized software that generated sounds closely resembling natural tinnitus. This is an improvement over a traditional clinical audiometer, which typically offers options like pure tones and white noise. Additionally, the accuracy of frequency detection for tinnitus was of 1 Hz.

Tinnitus sufferers with normal hearing do not show severe “disability.” In contrast, individuals with tinnitus who also have hyperacusis, as well as those with tinnitus and hearing loss, tend to have higher THI scores.

Patients with tinnitus matched to the 4000 Hz frequency reported a sudden onset of high-intensity tinnitus. Conversely, those experiencing tinnitus at frequencies of greater than 6000 Hz noted a gradual onset with a progressive increase in intensity.

DPOAE recording offers a quick and objective assessment of OHC function. This information is valuable and should be incorporated into the clinical evaluation of tinnitus sufferers with normal hearing to provide proper counseling and management.

## Figures and Tables

**Table 1:** The frequencies, F1 and F2, of the pure tones in DPOAE recordings (F2/F1 = 1.22).

**F2 (Hz)**	619	767	950	1000	1177	1458	1500	1805	2000	2236
**F1 (Hz)**	507.37	628.68	778.68	819.67	964.75	1195.08	1229.5	1479.5	1639.3	1832.78
**F2 (Hz)**	2770	3000	3430	4000	4249	5263	6000	6518	8074	10,000
**F1 (Hz)**	2270.49	2459	2811.47	3278.68	3482.78	4313.93	4918.03	5342.62	6618.03	8.196.72

**Table 2:** Results of the mean recorded DPOAEs in TG and CG; calculation of the mean differences between the two groups with 95% CI and *p*-values of the *t*-test. The symbol * indicates values with significant statistical differences (*t*-test, Bonferroni-corrected, *p* < 0.05). Abbreviations: DPOAEs = distortion product otoacoustic emissions; F2 = the frequency in Hz at which the DPOAEs were measured; TG = tinnitus group; CG = control group; N = number of participants in each group; SD = standard deviation; CI = coefficient interval.

DPOAEs’ Frequency Recording (F2, Hz)	Group	N	Mean DPOAEs’ Amplitude	SD	Mean Difference	95% CI		*p*-Value	Bonferroni-Corrected *p*-Value
						Lower	Upper		
619	TG	64	3.90	3.49	−4.46	−6.23	−2.70	<0.001	<0.001 *
	CG	20	8.36	3.37					
767	TG	64	2.42	6.31	−5.33	−8.27	−2.39	0.001	0.009 *
	CG	20	7.75	3.44					
950	TG	64	5.31	7.38	−4.72	−8.08	−1.35	0.007	0.14
	CG	20	10.03	2.73					
1000	TG	64	5.79	3.92	−4.57	−6.57	−2.57	<0.001	<0.001 *
	CG	20	10.36	3.91					
1177	TG	64	8.09	5.79	−2.69	−5.51	0.13	0.061	1.22
	CG	20	10.78	4.58					
1458	TG	64	8.28	6.77	−3.82	−6.90	−0.73	0.016	0.32
	CG	20	12.09	2.53					
1500	TG	64	8.23	5.79	−3.32	−5.98	−0.67	0.015	0.3
	CG	20	11.56	2.40					
1805	TG	64	6.60	8.40	−3.15	−6.95	0.66	0.10	2.0
	CG	20	9.75	2.71					
2000	TG	64	5.71	6.81	−2.34	−5.49	0.81	0.143	2.86
	CG	20	8.06	3.36					
2236	TG	64	3.50	7.37	−2.77	−6.12	0.57	0.103	2.06
	CG	20	6.27	2.42					
2770	TG	64	2.77	4.90	−0.70	−3.10	1.70	0.639	12.7
	CG	20	3.47	3.97					
3000	TG	64	2.97	6.27	−1.30	−4.23	1.64	0.563	11.26
	CG	20	4.27	3.63					
3430	TG	64	1.78	7.98	−3.36	−7.01	0.29	0.383	7.6
	CG	20	5.14	3.29					
4000	TG	64	1.11	7.08	−4.72	−8.02	−1.42	0.071	1.42
	CG	20	5.83	3.87					
4249	TG	64	−1.97	9.07	−7.15	−11.32	−2.99	0.006	0.12
	CG	20	5.18	3.93					
5263	TG	64	−5.22	10.98	−10.11	−15.15	−5.07	0.001	0.02 *
	CG	20	4.89	4.69					
6000	TG	64	−13.4	11.59	−16.76	−22.11	−11.42	<0.001	<0.001 *
	CG	20	3.33	5.40					
6518	TG	64	−11.0	11.58	−14.01	−19.39	−8.64	<0.001	<0.001 *
	CG	20	2.99	5.90					
8074	TG	29	−17.7	9.20	−16.99	−21.29	−12.68	<0.001	<0.001 *
	CG	20	−0.72	5.24					
10,000	TG	64	−15.4	8.10	−15.36	−19.61	−11.12	<0.001	<0.001 *
	CG	20	−0.10	9.04					

**Table 3:** Characteristics of TG participants in relation to abnormal DPOAEs at specific F2 frequency areas. The symbol * indicates values with significant statistical difference (Fisher’s exact Chi-square test, *p* < 0.05). (Abbreviations: TG= tinnitus group; DPOAEs = distortion product otoacoustic emissions; THI = Tinnitus Handicap Inventory).

Characteristics of TG Participants (64 Ears)		Abnormal DPOAEs, ≤1000 Hz		Abnormal DPOAEs, ~4000 Hz		Abnormal DPOAEs, ≥6000 Hz	
**Tinnitus onset**	Gradual	2 (3.1%)	*p* = 0.06 *	0	*p* < 0.001 *	43 (67.18%)	*p* < 0.001 *
	Sudden	4 (6.25%)		9 (14.0%)		6 (9.37%)	
**Tinnitus intensity self-assessment**	<70	2 (3.1%)	*p* = 0.06	3 (4.68%)	*p* = 0.17	45 (70.3%)	*p* = 0.002 *
	>70	4 (6.25%)		6 (9.37%)		4 (6.25%)	
**Degree of disability**	ΤHΙ < 58	6 (9.37%)	*p* = 0.031 *	2 (3.1%)	*p* = 0.025 *	29 (45.3%)	*p* = 0.456
	ΤHΙ > 58	0		7 (10.9%)		20 (31.25%)	

## Data Availability

The datasets presented in this article are not readily available because the data are part of an ongoing study.

## Abbreviations

The following abbreviations are used in this manuscript:

ABR	Auditory Brainstem Response
AAO	American Academy of Otolaryngology
CG	Control group
DPOAEs	Distortion product otoacoustic emissions
HNS	Head and Neck Surgery
IHCs	Inner Hair Cells
CNS	Central nervous system
MML	Minimum masking level
OAEs	Otoacoustic emissions
OHCs	Outer hair cells
TG	Tinnitus group
QR-code	Quick Response Code
SD	Standard deviation
SFR	Spontaneous Firing Rate
SNR	Sound-to-noise ratio
SPSS	Statistical Package for the Social Sciences
TEOAEs	Transiently evoked otoacoustic emissions
THI	Tinnitus Handicap Inventory
THI-G	Tinnitus Handicap Inventory—Greek version
